# Supplementary material for: Hidden risks associated with occupational pesticide exposure in women with breast cancer: High frequency of the Luminal B molecular subtype and occurrence of poor prognostic features
Source: PLoS One. 2026 Feb 5;21(2):e0339471. doi: 10.1371/journal.pone.0339471 (PMC12875504; doi:10.1371/journal.pone.0339471)
Supplement: S1 File — S1 Table. Association between molecular subtypes and clinical pathological variables with significance in breast cancer patients occupationally exposed to pesticides. S2 Table. Association between molecular subtypes and clinical pathological variables with significance in breast cancer patients unexposed to pesticides. (ZIP) [file pone.0339471.s001.zip › 03-S1_table_PONE-D-24-40245R6_FTC_final_2025-12-18.docx]

S1 Table - Association between molecular subtypes and clinical pathological variables with significance in breast cancer patients occupationally exposed to pesticides.

|  | Lum B | Lum A | Ratio Lum B/ Lum A | p-value | Lum B | HER2-amplified | Ratio Lum B / HER2-amplified | p-value | Lum B | Triple-negative | Ratio Lum B/Triple-negative | p-value |
| --- | --- | --- | --- | --- | --- | --- | --- | --- | --- | --- | --- | --- |
| Estrogen receptor expression | 65 | 58 | 1.12 | 0.53 | 65 | 10 | 6.5 | <0.01 | 65 | 0 | 0 | <0.01 |
| Progesterone receptor expression | 38 | 43 | 0.88 | 0.58 | 38 | 8 | 4.75 | <0.01 | 38 | 0 | 0 | <0.01 |
| HER2-amplified | 0 | 0 | 0 | NA | 0 | 24 | 0 | <0.01 | 0 | 0 | 0 | NA |
| KI67 (%) | 66 | 8 | 8.25 | <0.01 | 66 | 27 | 2.44 | <0.01 | 66 | 19 | 3.47 | <0.01 |
| Presence of angiolymphatic emboli | 18 | 15 | 1.2 | 0.6 | 18 | 6 | 3 | 0.01 | 18 | 13 | 1.38 | 0.37 |
| Lymph node invasion | 30 | 19 | 1.58 | 0.12 | 30 | 9 | 3.33 | <0.01 | 30 | 10 | 3 | <0.01 |
| Distant metastasis | 30 | 22 | 1.36 | 0.27 | 30 | 13 | 2.31 | <0.01 | 30 | 11 | 2.73 | <0.01 |
| Menopausal status | 46 | 42 | 1.1 | 0.67 | 46 | 14 | 3.29 | <0.01 | 46 | 25 | 1.84 | 0.01 |
| Pesticide exposure | 66 | 61 | 1.08 | 0.66 | 66 | 33 | 2 | <0.01 | 66 | 38 | 1.74 | <0.01 |
| Chemoresistance | 15 | 8 | 1.88 | 0.14 | 15 | 10 | 1.5 | 0.32 | 15 | 11 | 1.36 | 0.43 |
| Recurrence | 7 | 4 | 1.75 | 0.54* | 7 | 5 | 1.4 | 0.56 | 7 | 5 | 1.4 | 0.56 |

The columns with the molecular subtypes (Luminal A, Luminal B, HER2-amplified and Triple-negative) present the absolute frequency values ​​for each of the clinicopathological parameters. The ratio was calculated by dividing the absolute frequency of the 1st molecular subtype by the absolute frequency of the 2nd molecular subtype of the column title. The Chi-square test and Fisher's exact test were calculated using the comparison of the 1st and 2nd molecular subtypes reported in the ratio column arranged to the left of the p-value column. A p<0.05 was considered significant. NA = not applicable. The abbreviation Lum corresponds to the Luminal molecular subtype. α represents that the p-values ​​were calculated using Fisher's exact test, the other values ​​were calculated using the chi-square test for independence.
