# Supplementary material for: Hidden risks associated with occupational pesticide exposure in women with breast cancer: High frequency of the Luminal B molecular subtype and occurrence of poor prognostic features
Source: PLoS One. 2026 Feb 5;21(2):e0339471. doi: 10.1371/journal.pone.0339471 (PMC12875504; doi:10.1371/journal.pone.0339471)
Supplement: Attachment — Submitted filename: 03-Response_to_Reviewers_2025-02-19.docx [file pone.0339471.s004.docx]

Dear Dr. Sauli.

We would like to thank you for your analysis, criticism and considerations, as well as those of the reviewers, regarding our work for submission to this prestigious journal. Below are the responses to each point raised which, we hope, can address all criticisms of the manuscript.

Journal Requirements:

When submitting your revision, we need you to address these additional requirements.

1. Please ensure that your manuscript meets PLOS ONE's style requirements, including those for file naming. The PLOS ONE style templates can be found at

[https://journals.plos.org/plosone/s/file?id=wjVg/PLOSOne_formatting_sample_main_body.pdf](https://journals.plos.org/plosone/s/file?id=wjVg/PLOSOne_formatting_sample_main_body.pdf) and

[https://journals.plos.org/plosone/s/file?id=ba62/PLOSOne_formatting_sample_title_authors_affiliations.pdf](https://track.editorialmanager.com/CL0/https:%2F%2Fjournals.plos.org%2Fplosone%2Fs%2Ffile%3Fid=ba62%2FPLOSOne_formatting_sample_title_authors_affiliations.pdf/1/010f0194d4eb12b2-70f11606-bbc2-400d-b101-2f492457fd1d-000000/FzkjhRpjDZooW5sEHdGkHiOtZ04RrF2fyc9r9FBmiUk=196).

R.: The work was reviewed and formatted following the journal's style guides.

2. We note that the grant information you provided in the ‘Funding Information’ and ‘Financial Disclosure’ sections do not match.

When you resubmit, please ensure that you provide the correct grant numbers for the awards you received for your study in the ‘Funding Information’ section.

R.: Information regarding the financing of the work has been reviewed and corrected.

3. Thank you for stating the following in the Acknowledgments Section of your manuscript:

“The author(s) declare that financial support was received for the research, authorship, and/or publication of this article. Part of this research was supported by Carlos Chagas Institute, Fiocruz/PR, Fundação Araucária (grant number 639/2022) and by Conselho Nacional de Desenvolvimento Científico e Tecnológico – CNPq (grants number 402364/2021-0, 441017/2023-1, and 305335/2021-9)”

We note that you have provided additional information within the Acknowledgements Section that is not currently declared in your Funding Statement. Please note that funding information should not appear in the Acknowledgments section or other areas of your manuscript. We will only publish funding information present in the Funding Statement section of the online submission form.

Please remove any funding-related text from the manuscript and let us know how you would like to update your Funding Statement. Currently, your Funding Statement reads as follows:

“The author(s) received no specific funding for this work.”

Please include your amended statements within your cover letter; we will change the online submission form on your behalf.

R.: The section has been corrected as per guidelines.

4. Your ethics statement should only appear in the Methods section of your manuscript. If your ethics statement is written in any section besides the Methods, please move it to the Methods section and delete it from any other section. Please ensure that your ethics statement is included in your manuscript, as the ethics statement entered into the online submission form will not be published alongside your manuscript.

R.: Information regarding the ethics committee has been edited as requested.

Review Comments to the Author

Reviewer #1: Dear authors,

The article is good, but some explanations are needed.
The main problem is numerical errors. The numbers in the tables and in the text are different. The main conclusion is not consistent with the numerical values presented.
I suggest a review of the statistical analysis performed. Below are the main questions, requests and suggestions.

R.: We thank the reviewer for the work of analyzing and pointing out criticisms for the work, which benefits from the corrections. The entire manuscript was reviewed to correct inconsistencies, and we believe that all points were addressed. To ensure the information is accurate, all statistical analyses have been reviewed and the results remain correct.

Important questions.

Question 1 – The study article is about the existence of a statistical correlation between pesticides and breast cancer. There is a sample of 386 patients with breast cancer, of which 208 patients were exposed to pesticides, while 141 patients were not exposed to pesticides. In this count is missing 37 patients, probably without this information (missing value).

Line 206-207: “..After excluding patients with benign lesions, 386 patients
207 having a breast cancer diagnosis were included in the study..”
Line 226-227: “..Based on this, patients were categorized as occupationally exposed (n = 208) or unexposed to pesticides (n = 141).

Exposed patients (n = 208) + Unexposed patients (141) = 349 patients ≠ Total of patients (386). It is necessary to explain this mathematics.

R.: We apologize for this inconsistency in the final patient count. In an initial check of the database of 386 patients, we noted inconsistencies in the data, so 37 of the patients were removed, for a total of 349. The above information has been included in the manuscript on page 7, line 193. The patient counts have been corrected where necessary.

Question 2 – Lines 88-90. “..A total of 386 women were included in the study. After a structured interview, women were categorized as exposed (n=208) or unexposed (n=141) to pesticides..”

If this study is about whether there is a statistical correlation between pesticides and breast cancer, shouldn't it only consider the 349 patients classified as exposed or not exposed to pesticides?

R.: As in the previous answer, the information has been reviewed and corrected at this point as well. The above information has been included in the manuscript on page 7, line 193. The total patient figures have been corrected where necessary.

Question 3 – If this study is about whether there is a statistical correlation between pesticides and breast cancer, why consider patients about whom we do not have this information?

What is the purpose of including these 37 patients without classification regarding pesticide exposure?

R.: We appreciate your noting the inconsistency and we really have no reason to include them. Patients without sufficient information for analysis were removed from the total and were not considered in the initial analysis of the study. Their removal was indicated on page 7, line 193.

Question 4 – Line 307: “..the Luminal B molecular subtype was prevalent (32.83%),..”
Luminal B molecular subtype values in Table 1 (33.33%) and in line 307 (32.83%) are different. Furthermore, the Luminal B molecular subtype was NOT prevalent in Table 1. Explain.

R.: We appreciate the observation and the values in the text were corrected to match the data obtained, which are contained in Table 1. Regarding prevalence, the comparison made here was within the same group of exposed patients, thus, Luminal B (33.33%) was the most prevalent molecular subtype, when compared with Luminal A (30.81%), Her2-amplified (16.67%) and Triple-negative (19.19%). We chose to perform an intragroup analysis initially to maintain the consistency of the patients' conditions. This information was reinforced on page 10, line 311 of the manuscript. In the unexposed patients, the most prevalent was Luminal A (37.78%).

Question 5 – Problem: Values in Table 1 ≠ values in text. Correct the values presented
Line 299: ‘..33.8% as Luminal B,..” Table 1 ⎝ 34.37%

Line 300: “..16.34% as Triple-Negative..” Table 1 ⎝ 15.77%

Check the other numbers.

R.: We appreciate the corrections. The values have been corrected in the text according to Table 1.

Question 6 – line 368-369. Statement inconsistent with the values presented in Table 1.

“..and predominance of the molecular subtype Luminal B..”
R.: Following the answer above, this prevalence is intragroup, which maintains the consistency of the patients' conditions. This information was reinforced in the line.

Question 7 - line 383-384: Correct the values presented
“..In the exploratory analysis, a higher prevalence of patients with the Luminal B molecular subtype (33.8%) was identified..”

R.: The value was corrected in the text according to Table 1.

General questions

1 – Check in PLOS ONE whether reference citations should be placed before or after the punctuation marks. Example: “..carcinogenic potential,3,4 ..” or “..carcinogenic potential3,4,..”

R.: The formatting regarding citations was all reviewed in accordance with the journal's policies.

Other questions, requests, and suggestions.

Line 101 . Deleted: to breast cancer

“..compared to patients not exposed to breast cancer. Conclusions: These findings..”

Line 116-117. Normalmente a citação de referência pertence ao texto. Verifique o estilo da PLOS ONE.

“..carcinogenic potential,3,4 endocrine disrupting properties,5 genotoxicity, 6–8 and immunotoxicity.9–12,..”

Suggestion: Comma after reference

“..carcinogenic potential3,4, endocrine disrupting properties5, genotoxicity6–8, and immunotoxicity9–12,..”

Line 117. Change “..immunotoxicity.9–12,..” to “immunotoxicity,9–12.. or immunotoxicity9–12,..”

Line 125. Change “..as the European Union..” to “..as in the European Union..”

Line 126-127. Deleted: of women

“..where they represent 45 % of women, and certain regions..”

Line 129. Delete: breast

“..the incidence of breast cancer in women..”

R.: We thank the reviewer for the detailed analysis and all points unaddressed here have been corrected.

Reviewer #2: Dear authors,

The results described in the article relate to epidemiological studies and are extremely relevant.

There is no doubt that the effect of pesticides is reflected in the genetic status of the patient. Some mutations appear. I leave it to the authors' decision whether to include data on the characteristics and presence of some mutations specific to the region, or the presence of ethnic mutations. This would be very interesting.
R.: We thank the reviewer for the work of analyzing and pointing out criticisms of the work, which benefits from the corrections. We agree that the information on mutations is relevant and citations on the topic have been included on page 14, line 415, as follow.

We have previously investigated this question about the impact of pesticide exposure on the mutational landscape of these patients, which can be found in Scandolara TB, Valle SF, Teixeira CE, Scherer NM, de Armas EM, Furtado C, Boroni M, Jaques HDS, Alves FM, Rech D, Panis C, Bonvicino CR. Somatic DNA Damage Response and Homologous Repair Gene Alterations and Its Association With Tumor Variant Burden in Breast Cancer Patients With Occupational Exposure to Pesticides. Front Oncol. 2022 Jul 8;12:904813.

This study examined whether pesticide exposure influences mutational patterns in genes involved in homologous recombination and damage response in breast cancer patients. By analyzing tumor samples from 158 patients, we found that pesticide exposure was associated with more pathogenic mutations, particularly in those diagnosed before age 50 or carrying variants in BRCA1, BRCA2, or PALB2. These findings suggest that pesticide exposure may impact cancer development, mutational burden, and disease progression.

Comments:
1. Figure 1 lists all 27 municipalities. A similar list of these municipalities appears in lines 188-199. You need to remove this repetition from the text of the article.
R.: Thanks for the note. Names have been removed.

2. Subsection 2.1.2. Tumor characteristics, line 230, should be shortened, since all the data are given in the table.

R.: Thank you for your input. The information has been summarized and included as a Table.

Reviewer #4: This is a study of risks associated with occupational pesticide exposure. It includes 386 breast cancer patients from a region in Brazil known for high use of pesticides. A structured interview was performed, and clinical and pathology data were drawn from medical records. The authors conclude that pesticide exposure favors the occurrence of more aggressive breast cancer.

It is important to study the risks associated with pesticide use in agriculture among women in Brazil. Nevertheless, I have concerns regarding the work presented in this study.

R.: We thank the reviewer for the work of analyzing and pointing out criticisms of the work, which benefits from the corrections.

My main concern is the number of patients included, with only 208 exposed, and 141 unexposed patients.

Introduction:
The introduction explains the rationale for studying the association between pesticide use and breast cancer pathology.

Methods:
It says that patients were recruited from 2015 to 2017 (page 4, line 178). In the same page, line 182 it seems like the recruitment period was from 2015 to 2023.
It says that “this information could not identify individual participants” (page 4, line 178-179). What does that mean? Since medical records were used, individual patients would need to be identified.

R.: Thank you for your observation, the date has been corrected, 2015 to 2023, as stated on page 6, line 175. Regarding the statement that patients cannot be identified, this concerns the data made available in the publication of the manuscript, following the rules of the Ethics Committee, where the data used in the work cannot allow the identification of patients. The text was modified to make this statement clearer.

It is not necessary to name all the municipalities (page 4).

R.: The names of the cities were removed from the text.

Due to the high use of pesticides in this region in Brazil, are the authors sure that the unexposed group are truly unexposed to pesticides? The definition of “exposed” includes only highly exposed patients (living at least 50% of their lives under direct handling of such substances at least twice a week for every week of the year). By using such a strict definition, I would assume that you would miss many of the pesticide exposed patients. This needs to be addressed in the discussion.

R.: We agree that the informed criterion is the standard and is significant in pesticide exposure studies. However, in the present study we are concerned precisely with patients who are occupationally exposed and who, therefore, would not be considered exposed using the 50% direct exposure criterion. In a previous study by our group, da Silva et al. 2023. Brief research report pesticide occupational exposure leads to significant inflammatory changes in normal mammary breast tissue. Frontiers in Public Health, this criterion and its importance were discussed. This information was included in section Pesticide exposure assessment, page 7, line 198.

Starting with 389 patients, only 349 (208 + 141) seem to be included. Yet, looking at table 1, n=353.

R.: Regarding the value n=353 in Table 1, we clarified throughout the manuscript that the total number of patients was 349, including exposed (208) and unexposed (141). The mean age between exposed and unexposed patients is lower than this, 197+134=331, since we do not have data for all the 349 patients in the database. These values were reanalyzed and the statistics of the study were not modified.

With regards to tumor characteristics, section 2.1.2: The authors need to describe more thoroughly regarding the biomarkers. For instance, ER and PR are probably assessed by immunohistochemistry (IHC). It is stated that ER and PR >0 is defined as positive, but according to ASCO/CAP guidelines, a cut-off of 1% is used. Regarding HER2 it says “values of 3+…” This refers to HER2 protein expression assessed by IHC. The authors must separate between gene copy number (amplification), assessed by in situ hybridization, and protein expression, assessed by IHC. The choice of cut off (14%) for Ki67 should also be discussed.
With regards to disease characteristics: The risk stratification for death and recurrence should be explained, not only refer to a paper. What was the follow up from diagnosis? How was chemoresistance defined?

R.: Thanks for the feedback on the information. We have added a table to replace the information in the text and added the criteria that were requested here.

Results:
Heading 3.1. “Breast cancer patients occupationally exposed to pesticides have a prevalence of the Luminal B molecular subtype”. This sentence does not make sense.

R.: The sentence was corrected to: Breast cancer with prevalence of the Luminal B molecular subtype was observed in occupationally exposed to pesticides patients.

A table should not include only statistically significant results. It is stated that “other statistical values analyzed are present in the supplementary materials”.
The way the results are presented makes it very hard for the reader to follow. Example: “Increased frequency of Luminal B tumors was observed in relation to HER2-amplified (p=<0,01 and ratio= 2.31) and Triple-negative (p=<0,01 and ratio 2,73) in the exposed group (Table 2) regarding distant metastasis. So, in one sentence, molecular subtype, exposure status and distant metastasis are addressed. The same applies to the next sentence, and the way the tables are organized.

R.: We agree with the reviewer's statement that the data related to patient information is complex. In this article, we sought to correlate, through this form of multiparametric analysis, the complexity of the breast cancer scenario, a disease that is influenced by multiple factors. Additionally, the risk and residence determination ordinance, on which the work is based, uses this multiparametric approach to classify patients, which is a central problem of the manuscript. In an attempt to facilitate this interoperability and general understanding, changes were made throughout the work as a whole.

In Figure 1, the cases that are neither exposed nor unexposed are not presented. Not all 389 patients are included (208 + 141=349).

R.: We apologize for this inconsistency in the final patient count. In an initial check of the database of 386 patients, we noted inconsistencies in the data, so 37 of the patients were removed, for a total of 349. The above information has been included in the manuscript on page 7, line 193. The patient counts have been corrected where necessary.

In the tables, the numbers don’t add up, and some numbers are missing. Example: n=353 in Table 1, not 349. It says in the subtext that some variables are missing, but when only the “positive” number is given, it is not possible for the reader to see the number of missing values. For example, both ER positive and ER negative should be listed in the table. Why is age missing? When data is collected from medical records, I would assume age is available for all. The distribution of molecular subtypes varies depending on age. Whether the cancer was clinical or detected by mammography could also affect the distribution of molecular subtypes. This information is missing.

R.: This problem concerns the fact that not all patients presented all the information regarding the values we need in the study. This clarification was added to the table header. Specifically regarding age, and to justify the lack of data, the medical records accessed in the initial years of the study were accessed through interviews with the patients themselves. In some situations, the patients needed to report the age at diagnosis and this proved to be inconsistent with the medical information. In these cases, we chose not to use this information.

Discussion:
Strengths and weaknesses should be discussed more thoroughly. The strength mentioned in page 13, line 452-455 is not really a strength.

R.: We respectfully disagree with the reviewer on this point, since, to our knowledge, this is the first study to demonstrate that exposure to pesticides is a significant factor in the prognosis of the disease. We demonstrated that these patients, despite developing a tumor of the Luminal B molecular subtype, commonly associated with a milder pathology, have worse outcomes. This can be considered a strength of the work.

Last paragraph, page 12: This is more introduction-like.

R.: The paragraph was transferred to introduction.

The paragraph in page 13, line 444-450: The conclusion is not sound.

R.: We respectfully disagree with the reviewer on this point, since, to our knowledge, this is the first study to demonstrate that exposure to pesticides is a significant factor in the prognosis of the disease. We demonstrated that these patients, despite developing a tumor of the Luminal B molecular subtype, commonly associated with a milder pathology, present worse outcomes. This statement is consistent with the findings and the statistics used during the study.

Conclusion:
It is not true that the Luminal B subtype is associated with a good prognosis (page 13, line 457-458).

R.: We agree that the statement could be understood as overstated. We changed it to "commonly associated with a milder pathology".

General comments:

Several sentences are not precise enough. For example: “This trend towards a worse prognosis of women….” (page 11, line 379-380). Breast cancer must be mentioned for this sentence to make sense.

R.: We appreciate the observation and the work has been revised to correct these impressions.

The manuscript needs to be revised due to several grammatical errors and spelling mistakes. Example: “The patients were recruitment….” (page 4, line 176-177) and “…, one probable reason why do he have a prevalence…”. (page 11, line 391).

R.: We appreciate the observation and the work has been revised to correct these impressions.

It is important to distinguish associations and causality. An association does not necessarily mean that there is causality. This is very important and must be addressed more thoroughly.

R.: We agree with the reviewer and the explanation has been added as a weakness of the work on page 16, line 462. It is important to remember that observational correlation does not necessarily imply causality, as a limitation of any exploratory work.

Reviewer #5: I have gone through the manuscript titled "Hidden risks associated with occupational pesticide exposure in women with breast cancer: high frequency of the Luminal B molecular subtype and occurrence of poor prognostic features".
I have the following observations regarding the manuscript:

R.: We thank the reviewer for the work of analyzing and pointing out criticisms of the work, which benefits from the corrections.

1.The study lacks some clinical applications perspectives. Although the study identifies an association between pesticide exposure and breast cancer, it does not adequately discuss the causal mechanisms between exposure and tumor progression or immune dysregulation.

R.: We agree with the reviewer on this point, however, it was not the aim of this publication to comprehensively study the causality of what was observed in this population.

Concerning the clinical application perspectives, we added the following to the final paragraph of discussion/conclusions:

These findings are clinically relevant since we demonstrated the urgent need for targeted healthcare strategies for women exposed to pesticides, particularly in rural areas with high pesticide use such as Brazil. Our data suggest that pesticide exposure may be linked to more aggressive forms of breast cancer, with worse prognoses including increased recurrence, chemoresistance, and metastasis. These results highlight the importance of early detection and personalized treatment approaches for pesticide-exposed individuals, potentially guiding clinicians to monitor for aggressive disease progression and tailor therapeutic regimens accordingly.

Also, we included information about the pesticide mechanisms implicated in breast cancer generation (page 4, line 106-129):

Pesticides can elevate the risk of breast cancer through various mutagenic and nonmutagenic mechanisms, acting either directly as carcinogens or indirectly as biochemical modifiers and hormonal disruptors. The underlying mechanisms include endocrine disruption, genotoxicity, epigenetic alterations, enhanced cell migration, invasion, and stemness, angiogenesis, and tumor growth, among others.

Glyphosate is an endocrine disrupting substance, a pivotal mechanism proposed to breast carcinogenesis. In normal mammary cells, it promotes estrogenic activity by mimicking 17β-estradiol (E2), activating estrogen receptor α (ERα), leading to phosphorylation, degradation, and increased transcriptional activity, as well as stimulating cell proliferation and growth, even at very low concentrations. In breast cancer cells, glyphosate exposure leads to altered expression of cell proliferation-related genes, and dysregulation of key genes involved in tumor aggressiveness and metastasis, such as HIF-1.

Atrazine, also exhibit endocrine-disrupting effects, particularly on mammary gland development in murines, delaying mammary gland development and increasing steroidogenesis and hormone levels. Hormone deregulation is also documented for glyphosate and atrazine human exposures. In breast cancer cells, atrazine alters protein expression and modulates antioxidant defense gene expression, promoting genomic instability and oxidative stress-induced damage, a recognized mechanism for breast cancer development and progression, also linked to immune deregulation in patients and inflammatory changes in normal mammary tissue in exposed women.

2.The current exposure standard requires at least two exposures per week, every week of the year, which is a broad criterion. It does not thoroughly consider that different types of pesticides (e.g., carcinogenic or endocrine-disrupting) may have different health impacts, particularly the effects of exposure dose and exposure type.

R.: We agree that the informed criterion is the standard and is significant in pesticide exposure studies. However, in the present study we are concerned precisely with patients who are occupationally exposed and who, therefore, would not be considered exposed using the 50% direct exposure criterion. In a previous study by our group, da Silva et al. 2023. Brief research report pesticide occupational exposure leads to significant inflammatory changes in normal mammary breast tissue. Frontiers in Public Health, this criterion and its importance were discussed. This information was included on section Pesticide exposure assessment, page 7, line 205.

3.There is a lack of long-term follow-up data. It is recommended to increase the sample size and conduct long-term follow-up studies to better understand the long-term effects of pesticide exposure.

R.: We agree with the reviewer on this point, which is directly addressed in the discussion as a possible improvement to the work. However, given the specificity of this population, which is precisely why it is neglected in surveys related to severity, we believe that our work can shed light on a population and that, precisely with this, more cases can be observed, thus reinforcing or refuting the findings presented here.

Based on these observations, I would like to give a 'major revision' of the manuscript as decision. All the best to the authors!

Regards

Reviewer #6: The manuscript "Hidden risks associated with occupational pesticide exposure in women with breast cancer: high frequency of the Luminal B molecular subtype and occurrence of poor prognostic features" investigated the impact of chronic and continued occupational pesticide exposure on the clinicopathological profiling of breast cancer, with the aim of determining a clinicopathological signature associated with pesticide exposure.

R.: We thank the reviewer for the work of analyzing and pointing out criticisms of the work, which benefits from the corrections.

This study presents a better comprehension about the pesticides harmful in relation to the clinicopathological characteristics of tumor, as molecular subtype, grade, average tumor size, presence of angiolymphatic emboli, lymph node positivity and distant metastasis, besides the characteristics of the disease, as stratification of the risk of death and recurrence, recurrence profile, chemoresistance to treatment and occurrence of death. In this sense, the topic investigated in the study is relevant to environmental, health and oncology. Furthermore, this is a clinical study that can help medical decisions about the best treatment protocols when considering the patient exposition or not to pesticides. Finally, the study is important to alert the population about the dangers and impacts caused by pesticides and to assist public health in the sense of prevention.

The introduction shows clearly the importance to study pesticides and breast cancer e also presents the novelty and the objective of the study. Besides, methods section is described satisfactorily and presents all necessary points to the study comprehension. Table 1 reinforce every method applied in the study. The discussion also demonstrates justificatives of the results and comparison with the literature. However, results section need authors attention, because the data showed have not mathematical concordance and need major review.

In view to improve the manuscript quality some appointments are listed below:
R.: Thank you for your careful observation.

1. Keywords: the words already present in the title should be changed by other words, in view of increase the possibilities of manuscript search. For example: pesticides by agrochemicals and breast cancer by breast tumor or breast neoplasm.
R.: We appreciate your comments and have modified the text.

2. Line 186 in the number 500,000 inhabitants the comma need be changed by point.
R.: The typo has been fixed.

3. Table 1 subtitle - the following municipalities should maintain the original language of the name: 16 - Pérola D' west -> Pérola D'Oeste; 20 - Royalty -> Realeza; 21 - Renaissance - Renascença.

R.: The typo in citys’s names has been fixed.

4. Table 1 data and their results presented in the text need major review. For examples:
- Total population presented is 386, but the population exposed is 208 and not exposed is 141, however, 208 + 141 = 349 and not 386.

- Mean age of 353 patients – 197 exposed and 134 not exposed, but 197 + 134 = 331 and not 353.

- Menopausal status of 229 patients – 130 exposed and 89 not exposed, but 130 + 89 = 219.

- BMI of 259 patients – 150 exposed and 97 not exposed, but 150 + 97 = 247.
- Luminal A 33.24% (n=118) 30.81% (n=61) 37.78% (n=51), but 61 + 51 = 112 and not 118.

- Luminal B 34.37% (n=122) 33.33% (n=66) 34.81% (n=47), but 66 + 47 = 113 and not 122.

- HER2-amplified 16.62% (n=59) 16.67% (n=33) 15.56% (n=21), but 33 + 21 = 54 and not 59.

- Triple-negative 15.77% (n=56) 19.19% (n=38) 11.85% (n=16), but 38 + 16 = 54 and not 56.

- Presence of angiolymphatic emboli 25.52% (n=86) 27.51% (n=52) 24.06% (n=32), but 52 + 32 = 84 and not 86.

... other...

If there is a equivocate mathematical it need be adjusted and if there is not this difference need be explicated. Besides, the percent of these data also may be equivocated, since the number of exposed and not exposed patients would be not correct.

R.: We appreciate the observation and detailed analysis of the data. In fact, we initially included 37 patients in the study, including exposed and non-exposed patients, who did not have all the variables of the study. This lack of information is due to the fact that some of the patients' medical records were incomplete and thus made it difficult to obtain information. Therefore, we decided to remove the patients from the study and thus promote a better understanding of the findings. All the mathematics and statistics of the study were reviewed, and the results continue to support the conclusions of the study.

5. The other next tables may present data also equivocate, in view of the difference in the calculi, as described above. Moreover, the abstract results, the results section and the discussion of the results also be compromited.

R.: We thank you again for your comments. With the changes discussed in the previous question, we have corrected the inconsistencies also pointed out here. All the mathematics and statistics of the work were reviewed and the results continue to support the conclusions of the study.

6. NA described in tables 3 and 4 needs to be presented in full at their respectives subtitles.

R.: The information was inserted.

7. Insert p value in table 1 in order to better comprehension of the table, without necessary to read complementary text results.

R.: We understand and agree that this suggestion may make the table easier to read, but we respectfully chose not to include the information in this way. The table is used to extract intragroup information, for example, to demonstrate the differences between molecular subtypes only in exposed patients. Therefore, we understand that including this information may confuse the reader.

6. PLOS authors have the option to publish the peer review history of their article ([what does this mean?](https://track.editorialmanager.com/CL0/https:%2F%2Fjournals.plos.org%2Fplosone%2Fs%2Feditorial-and-peer-review-process%23loc-peer-review-history/1/010f0194d4eb12b2-70f11606-bbc2-400d-b101-2f492457fd1d-000000/DNhyv-qt4QOvFWkEGaW1F8yYy2dhp1t-yxgYYDmM-fU=196)). If published, this will include your full peer review and any attached files.

If you choose “no”, your identity will remain anonymous but your review may still be made public.

**Do you want your identity to be public for this peer review?** For information about this choice, including consent withdrawal, please see our [Privacy Policy](https://track.editorialmanager.com/CL0/https:%2F%2Fwww.plos.org%2Fprivacy-policy/1/010f0194d4eb12b2-70f11606-bbc2-400d-b101-2f492457fd1d-000000/aXHpntXjcxph5VB4wcPmmTzPGlOfyhHqCoBQNbhF3wI=196).

Reviewer #1: **Yes:** Paulo Laerte Natti

Reviewer #2: No

Reviewer #4: No

Reviewer #5: No

Reviewer #6: No

[NOTE: If reviewer comments were submitted as an attachment file, they will be attached to this email and accessible via the submission site. Please log into your account, locate the manuscript record, and check for the action link "View Attachments". If this link does not appear, there are no attachment files.]

While revising your submission, please upload your figure files to the Preflight Analysis and Conversion Engine (PACE) digital diagnostic tool, [https://pacev2.apexcovantage.com/](https://track.editorialmanager.com/CL0/https:%2F%2Fpacev2.apexcovantage.com%2F/1/010f0194d4eb12b2-70f11606-bbc2-400d-b101-2f492457fd1d-000000/G074acfmUbM-xflCmBsdn6k0IL9UIEhUY5l12OaStVc=196). PACE helps ensure that figures meet PLOS requirements. To use PACE, you must first register as a user. Registration is free. Then, login and navigate to the UPLOAD tab, where you will find detailed instructions on how to use the tool. If you encounter any issues or have any questions when using PACE, please email PLOS at [figures@plos.org](mailto:figures@plos.org). Please note that Supporting Information files do not need this step.
