# Supplementary material for: Hidden risks associated with occupational pesticide exposure in women with breast cancer: High frequency of the Luminal B molecular subtype and occurrence of poor prognostic features
Source: PLoS One. 2026 Feb 5;21(2):e0339471. doi: 10.1371/journal.pone.0339471 (PMC12875504; doi:10.1371/journal.pone.0339471)
Supplement: Attachment — Submitted filename: 03-Response_to_Reviewers_2_2025-04-02.docx [file pone.0339471.s006.docx]

**PLOS ONE**

**Title:** Hidden risks associated with occupational pesticide exposure in women with breast cancer: high frequency of the Luminal B molecular subtype and occurrence of poor prognostic features

**Manuscript ID:** PONE-D-24-40245R1

I have read all the questions from reviewers 1, 2, and 6 and believe that the authors have answered them satisfactorily and enlighteningly. In general, the issues they raised covered the major points of the manuscript.

They argued satisfactorily on the issues where the authors disagreed with reviewer 2.

I have only a few minor points to suggest:

**Minor points**

-Line 61: Remove “in Brazilian south). The region has already been described at the beginning of the paragraph.

R.: Change applied.

-I suggest including the p-value in significant results.

R.: Change applied.

Line 77: “neoplasm ;”. Remove space between neoplasm and the comma.

R.: Change applied.

Keywords: I suggest changing the word recurrence that already appears in the text to relapse.

R.: Change applied.

Line 107: I suggest including a sentence saying that the southeastern region of northern Paraná has a prevalence of glyphosate and atrazine use. This sentence would link to the presentation of the three pesticides in the next paragraph. Why was 2,4-dichlorophenoxyacetic (2,4-D) not included in the Introduction?

R.: Yes, we agree that this information is important and we have included a paragraph in the introduction, line 127.

Line 111: “cell proliferation and growth],(“. Please, remove the bracket.

Like #reviewer1, I found the presentation of references in the text strange. I checked the publications and found a different format. Please review (<https://journals.plos.org/plosone/article?id=10.1371/journal.pone.0297048>)

R.: Change applied.

Line 146: I suggest including that the population is specific to the Southwest region of Paraná, Brazil.

R.: Change applied.

Line 235: "dichotomized as presence and absence". This sentence is confusing to me. I don't understand how the data on age at diagnosis and menopause were dichotomized. Would it be in exposed and unexposed women, instead of presence and absence?

R.: The dichotomized as presence and absence is only in the menopause variable. The text has been modified to make this explicit.

Lines 239-250: I suggest merging this data with the data from the Pesticide exposure assessment item. The information in the "Patient characteristics" item is sometimes repeated and concerns the same subject.

R.: Change applied.

Line 253: “characteristics :” removes the space between characteristics and colon.

R.: Change applied.

Line 375: This sentence is correct: clinical behavi(57)or.(56). Please check.

R.: The correct is and, not or. Change applied.

Line 415: Genes should be written in italics: *BRCA1, BRCA2* or *PALB2*.

R.: Change applied.

Lines 443-447: In the study by Pizzatti et al. (2020)(58), did the authors evaluate TNF gene or protein expression? This information is essential. If it is, the gene should be written in italics.

R.: Change applied.

Table 1: Standardize the number of decimal places.

For example: 72.1% (n=261) 67.49% (n=137) 78.1% (n=107)

R.: Change applied.
