# Supplementary material for: Hidden risks associated with occupational pesticide exposure in women with breast cancer: High frequency of the Luminal B molecular subtype and occurrence of poor prognostic features
Source: PLoS One. 2026 Feb 5;21(2):e0339471. doi: 10.1371/journal.pone.0339471 (PMC12875504; doi:10.1371/journal.pone.0339471)
Supplement: Attachment — Submitted filename: Plos_One.docx [file pone.0339471.s005.docx]

Francisco Beltrão Cancer Hospital (Ceonc)

an Hospital Cancer Center that serves patients from western Parana Stage
