# Supplementary material for: Hidden risks associated with occupational pesticide exposure in women with breast cancer: High frequency of the Luminal B molecular subtype and occurrence of poor prognostic features
Source: PLoS One. 2026 Feb 5;21(2):e0339471. doi: 10.1371/journal.pone.0339471 (PMC12875504; doi:10.1371/journal.pone.0339471)
Supplement: Attachment — Submitted filename: 03-Response_to_Reviewers_3_Carol_2025-09-02.docx [file pone.0339471.s007.docx]

PONE-D-24-40245R2

Hidden risks associated with occupational pesticide exposure in women with breast cancer: high frequency of the Luminal B molecular subtype and occurrence of poor prognostic features.

Dear Dr. Sauli.

We would like to thank you for your analysis, criticism and considerations, as well as those of the reviewers, regarding our work for submission to this prestigious journal. Below are the responses to each point raised which, we hope, can address all criticisms of the manuscript.

Editor comments:

When responding to reviewer comments, the authors should indicate all limitations incurred in their study. The authors should also improve the methodology...including study design ..patients sampling.....methods...results and their respective discussion.

R.: The entire work has been reviewed, and the necessary changes have been made as requested and discussed in the point-by-point response. We believe that even after all the modifications, the results and conclusions were maintained, which reinforces the robustness of the publication and, therefore, we request a new evaluation.

Reviewer #7:

The authors have satisfactorily addressed all my comments/suggestions. Since in the first review I proposed only a "minor revision", I would like to point out that the manuscript is now ready for publication.

R.: We thank the reviewer for the previous notes and for his contribution to the work.

Reviewer #8:

The manuscript "Hidden risks associated with occupational pesticide exposure in women with breast cancer: high frequency of the Luminal B molecular subtype and occurrence of poor prognostic features" presents a study of how breast cancer subtypes distributes changes between exposed / non-exposed pesticide groups.

The article is clearly written and the analysis direct, and in a very relevant topic. My main comment is if there are not confounding variables, for instance education level / subject income / ancestry ? I'm worried exposure to pesticide could be highly correlated to some other variables.

If the authors have some data about that will be great, if not at least should be mentioned as a limitation.

R.: We appreciate the comment. Unfortunately, we do not have additional information such as those mentioned by the reviewer. This has been included as a limitation of the study (page 8): In the present dataset we do not have information regarding education level / subject income / ancestry, which may be a limitation of this work.

Some minor comments:

- Please check "2,4-D?"

R.: 2,4-dichlorophenoxyacetic. This was included in the work (page 3).

- Can you detail "Inconsistencies in the data?"

R.: Some of the variables did not contain complete values, or the values were out of scale, indicating collection error. This comment was included in the work (page 5).

Reviewer #9:

Hidden risks associated with occupational pesticide exposure in women with breastcancer: high frequency of the Luminal B molecular subtype and occurrence of poor prognostic features.

Title: Luminal B.

We observed limitations of information related to luminal B.

We have luminal B her negative and luminal B her positive.

Review it.

R.: We are sorry about the confusion. Luminal B subtype was considered when tumors had any positivity for ER and/or PR and ki67 index above 14%, without HER2 amplification. HER2-amplified tumors were considered when tumors had any ER/PR/ki67 value and amplification for HER2 confirmed by IHC followed by FISH test when necessary.

We revised this information to the Methods section Tumor characteristics, page 7.

Methods

1. Improve localization

R.: Information was included about Francisco Beltrão Cancer Hospital that is a public Hospital Cancer Center that attends patients from 27 municipalities of Parana state southwest, page 5.

2. Organize the information

- Images suggestive of breast lesions identified by mammograms and ultrasound were included

Evaluate breast lesions? Birads IV, V or Birads III, IV or V?

- Imagens suggestive of adnominal breast lesions (Birads IV or V ?) identified by mammograms and ultrasound were included

- Biopsy of suspicious lesion = Birads IV or V

R.: Unfortunately, we did not have direct access to breast images from patients. Only the clinicians had access to image when staging and stratifying patients.

3. inconsistencies in the data?

Related to the condition?

37/386 inconsistencies = 9.5%

37 (9.5%) of inconsistency – inform it

R.: Some of the variables did not contain complete values, or the values were out of scale, indicating collection error. This comment was included in the work (page 5).

4. comprehensive questionnaire with 61 questions

Consider it in supplementary material

If you used the 60 questions by the Thesis of Shaiane Carla Gaboardi, consider it methods

<https://sucupira.capes.gov.br/sucupira/public/consultas/coleta/trabalhoConclusao/viewTrab> alhoConclusao.jsf?popup=true&id_trabalho=10956083

R.: The questionnaire used in this study has already been published and is cited in the paper, as indicated on page 7, reference 57, Panis C, Gaboardi SC, Kawassaki ACB, Dias ECM, Teixeira GT, Silva DRP da, et al. Characterization of occupational exposure to pesticides and its impact on the health of rural women. Rev Ciênc Farm Básica E Apl. January 21, 2022;43:1–14. In this case, it would not be possible to include it in the supplementary material, as indicated by the reviewer.

5. Report a table showing all conditions related to the group considered exposed to pesticide

R.: This information is contained in the work table.

6. Despite similar geographic locations, genetic backgrounds, and cultural and dietary habits, the unexposed group had no occupational pesticide exposure.
genetic backgrounds and cultural habits

All patients were born in this region?

R.: Yes. The information was included in the page 6.

Do you have information about race?

If no, delete this information

R.: All patients were Caucasian. The information was included in the page 7.

7. Tumor characteristics

- Ann Oncol. 2023; Nov 1;34(11):970–86. The reference do not classify the tumors
- The publication is: Strategies for subtypes—dealing with the diversity of breast cancer: highlights of the St Gallen International Expert Consensus on the Primary Therapy of Early
Breast Cancer 2011. Annals of Oncology 2011; 22: 1736-1747

R.: We changed the reference, accordingly, thank you for suggesting.

8. Based on St Gallen consensus we have Luminal B Her positive.

You do not reported these patients?

He have: Luminal A, Luminal B her negative, Luminal B her positive, Triple negative and Her2 (triple positive)

R.: As stated in the methods section, all HER-amplified patients were grouped independently of their ER/PR/ki 67 status. We revised this information to the Methods section Tumor characteristics, page 7.

9. Patients were from 2015 to 2023. To evaluate recurrence the cutoff time must be in 2018 (five years).

To evaluate these conditions, we must have time and Kaplan Meier methods.
- Do not evaluate recurrence or survival. To evaluate this conditions, you have to report other informations as disease free-survival, cancer specific survival, mean follow up, status of the patients at the end of study.

- Consider a transversal study evaluating patients at diagnosis. No not evaluate death.
R.: We are sorry about this, we only considered if patients were dead or alive at the end of the follow-up. We do not have the exact date of death, since the medical records do not always have the precise information. Because of this, we do not have precise information concerning their disease free-survival, cancer specific survival, or mean follow up. For this reason, the death variable was removed from the work.

10. Disease characteristics

- Chemoresistance development: You considered RECIST

Patients were submitted to chemotherapy

Inform number or patients, type of chemotherapy and disease response

-Stratification for death and recurrence reference 59

The data is inconsistent This page no longer exists. This is an order to release APAC.
If you want to report chemoresistance, report information about tumor characteristics, chemotherapy and response. If no exclude this information

R.: Thanks for your comment. We added the following to the methods section, page 8:

Patients with early-stage breast cancer who underwent neoadjuvant chemotherapy and were subsequently followed for disease recurrence were included in the study. Individuals who developed recurrence or systemic progression received adjuvant therapy. Chemotherapy data were retrieved from the Authorization for Outpatient Procedures (APAC) system. The chemotherapy protocols administered to the patients included three main regimens: AC-T, CMF, and TCH. The AC-T protocol consisted of doxorubicin (60 mg/m², day 1) combined with cyclophosphamide (600 mg/m², day 1) every 21 days for four cycles, followed by paclitaxel (175 mg/m²) administered either weekly for 12 weeks or every 21 days after completion of the AC cycles. The CMF regimen comprised cyclophosphamide (600 mg/m², day 1), methotrexate (40 mg/m², day 1), and 5-fluorouracil (600 mg/m², day 1), repeated every 21 days for six cycles. Patients receiving the TCH regimen were treated with trastuzumab (loading dose of 8 mg/kg, followed by 6 mg/kg on day 1 every 21 days for 12 months), carboplatin (AUC 6, day 1 every 21 days for six cycles), and docetaxel (75 mg/m², day 1).

For patient classification, individuals who did not achieve a response to neoadjuvant chemotherapy were defined as chemoresistant. The assessment followed the RECIST (Response Evaluation Criteria in Solid Tumours, available at: <https://recist.eortc.org/recist-1-1-2/>) guidelines.

Imaging reports from mammography, diagnostic ultrasound, breast MRI, CT, and PET-CT during follow-up were analyzed by clinicians in accordance with RECIST recommendations to establish baseline and post-treatment status. For each patient, the same imaging modality was applied at baseline and follow-up to ensure consistency.

Treatment response was categorized as follows: complete response, defined as the disappearance of all target lesions; partial response, defined as at least a 30% reduction in target lesion size without evidence of new lesions; progressive disease, defined as a minimum 20% increase in lesion size relative to baseline and/or the appearance of new lesions in the breast or distant organs; and stable disease, defined as insufficient shrinkage to meet partial response criteria and insufficient increase to meet progression criteria. Patients were monitored over a five-year follow-up period. Based on these criteria, patients were classified as responsive (complete response) or chemoresistant (partial response, progressive disease, or stable disease).

Concerning the risk stratification for recurrence and death, patients were stratified into risk categories based on lymph node status, tumor size, histological grade, hormone receptor expression, HER-2 status, molecular subtype, and age. Low-risk patients had negative lymph nodes and met all of the following criteria: tumor size (pT) under 2 cm, histological grade 1, estrogen receptor (ER) or progesterone receptor (PR) positive, HER-2 negative, molecular subtype luminal A, and age ≥35 years. Intermediate-risk patients had negative lymph nodes but met at least one of the following criteria: tumor size >2 cm, histological grade 2–3, ER or PR negative, molecular subtype luminal B (HER-2 negative), age <35 years, or 1–3 positive lymph nodes if ER and PR were positive. High-risk patients included those with 4 or more positive lymph nodes, lymph node-negative tumors that were triple-negative (ER, PR, and HER-2 negative) with pT >2 cm, or lymph node-negative tumors with pT >1 cm and HER-2 positive status.

Results
- Your study appears to be well-done, but the characteristics of the patients and tumors are not reported, which prevents us from assessing the study's reproducibility. Also the criteria used to consider exposed/ unexposed.

- Please include this information in a supplementary table.

R.: Information regarding patient characteristics is shown in Table 01, while information regarding the determination of exposed and non-exposed patients is described in the Pesticide exposure assessment section, in materials and methods. We review and correct any inconsistencies.

- Clinical characteristics at diagnosis: Age, race, tumor mean size, TNM, T-TNM, N-TNM, M-TNM, molecular subtype (5 types),

R.: Information regarding patient age, tumor size and molecular subtype was shown in Table 01. The information about race was included in materials and methods, page 7.

- Treatment: neoadjuvant chemotherapy, response to chemotherapy; type of treatment, follow up, disease free-survival, sites of recurrence, overall specific survival, status at last follow up

R.: Thanks for your comment. We added the following to the methods section, page 8:

Patients with early-stage breast cancer who underwent neoadjuvant chemotherapy and were subsequently followed for disease recurrence were included in the study. Individuals who developed recurrence or systemic progression received adjuvant therapy. Chemotherapy data were retrieved from the Authorization for Outpatient Procedures (APAC) system. The chemotherapy protocols administered to the patients included three main regimens: AC-T, CMF, and TCH. The AC-T protocol consisted of doxorubicin (60 mg/m², day 1) combined with cyclophosphamide (600 mg/m², day 1) every 21 days for four cycles, followed by paclitaxel (175 mg/m²) administered either weekly for 12 weeks or every 21 days after completion of the AC cycles. The CMF regimen comprised cyclophosphamide (600 mg/m², day 1), methotrexate (40 mg/m², day 1), and 5-fluorouracil (600 mg/m², day 1), repeated every 21 days for six cycles. Patients receiving the TCH regimen were treated with trastuzumab (loading dose of 8 mg/kg, followed by 6 mg/kg on day 1 every 21 days for 12 months), carboplatin (AUC 6, day 1 every 21 days for six cycles), and docetaxel (75 mg/m², day 1).

For patient classification, individuals who did not achieve a response to neoadjuvant chemotherapy were defined as chemoresistant. The assessment followed the RECIST (Response Evaluation Criteria in Solid Tumours, available at: <https://recist.eortc.org/recist-1-1-2/>) guidelines.

Imaging reports from mammography, diagnostic ultrasound, breast MRI, CT, and PET-CT during follow-up were analyzed by clinicians in accordance with RECIST recommendations to establish baseline and post-treatment status. For each patient, the same imaging modality was applied at baseline and follow-up to ensure consistency.

Treatment response was categorized as follows: complete response, defined as the disappearance of all target lesions; partial response, defined as at least a 30% reduction in target lesion size without evidence of new lesions; progressive disease, defined as a minimum 20% increase in lesion size relative to baseline and/or the appearance of new lesions in the breast or distant organs; and stable disease, defined as insufficient shrinkage to meet partial response criteria and insufficient increase to meet progression criteria. Patients were monitored over a five-year follow-up period. Based on these criteria, patients were classified as responsive (complete response) or chemoresistant (partial response, progressive disease, or stable disease).

Improve Table one

Variable – caractheristics – exposed – unexposed – total – p

Organize variables. For example, molecular subtype is one variable; grade is one variable.

Disease characteristics – inconsistency data

R.: The information in Table 1 was provided in a format that allowed for differentiated analyses of the data. This format allows the reader to evaluate each characteristic (e.g., luminal A vs. luminal B) of each variable (e.g. molecular subtype vs grade), rather than just the entire variable. The way we organized the table also allows for comparisons of each characteristic against all others, both in patients exposed and/or unexposed to pesticides. Therefore, we respectfully maintain the formatting to maintain the structure of the analyses.

11- What the criteria used?

Of this population, 8.25% were stratified as low risk for 304 death and recurrence, 55.87% into intermediate risk, and 35.87% were classified 305 as high risk.

R.: The same criteria (Joint Ordinance No. 5 of April 18, 2019) were used for risk stratification in all population subdivisions used in the work.

12- Review molecular evaluation/ Luminal B Her positive/ Luminal B her negative
Regarding the molecular subtype, 33.24% of the patients were classified as Luminal A, 34.37% as Luminal B, 16.62% as HER2-amplified, and 15.77% as Triple-Negative.

R.: As stated in the methods section, all HER-amplified patients were grouped independently of their ER/PR/ki 67 status. We revised this information to the Methods section Tumor characteristics, page 7.

13. Follow Up? Condition of the patient at last follow up?

About 7% of the patients died, 9.36%of the patients had disease recurrence

R.: As explained in the previous question, the death variable was removed from the work.

14. Chemoresistance? At neoadjuvant chemotherapy, disease progression after chemotherapy?

18.97% of the patients developed chemoresistance.

R. : To better clarify this point, we used previous work by the group that describes chemoresistance in patients in detail. Alves, F. M., dos Santos Jaques, H., Orrutéa, J. F. G., de Oliveira Silva, A. G., Machado, M. G., Smaniotto, L. L., ... & Panis, C. (2024). Changes in systemic oxidative stress correlate to chemoresistance and poor prognosis features in women with breast cancer. Revista de Senología y Patología Mamaria, 37(3), 100598.

15. Luminal A versus Luminal B?

Luminal B her positive?

R.: As stated in the methods section, all HER-amplified patients were grouped independently of their ER/PR/ki 67 status. We revised this information to the Methods section Tumor characteristics, page 7.

16. Discussion is based of results. Review the methods and results

R.: The review was revised, when necessary, according to new modifications to the work.
