# Supplementary material for: Hidden risks associated with occupational pesticide exposure in women with breast cancer: High frequency of the Luminal B molecular subtype and occurrence of poor prognostic features
Source: PLoS One. 2026 Feb 5;21(2):e0339471. doi: 10.1371/journal.pone.0339471 (PMC12875504; doi:10.1371/journal.pone.0339471)
Supplement: Attachment — Submitted filename: 03-Response_to_Reviewers_4_2025-09-25.docx [file pone.0339471.s008.docx]

PONE-D-24-40245R2

Hidden risks associated with occupational pesticide exposure in women with breast cancer: high frequency of the Luminal B molecular subtype and occurrence of poor prognostic features.

Dear Dr. Sauli.

We would like to thank you again for your analysis, criticism and considerations. Below are the requested modifications.

Editor comments:

The authors have done significant improvement of this submission, which may warrant acceptance after minor corrections, which is to highlight methodological limitations of their work in the conclusion part. This may be done within a very short period of time (i give them 1 week) before this submission can be accepted.

R.: Thank you for your evaluation and as a response, we have inserted the following paragraph in the materials and methods section, in the topic Study Limitations.

This work has some limitations. We did not divide the subjects into five molecular subtypes, as the four subtypes we used were chosen because they were the most prevalent in our cohort. All Luminal B patients are HER-positive, we do not have Luminal B her-negative in our cohort. The fifth subtype was excluded due to its low incidence. We used only the RECIST (Response Evaluation Criteria In Solid Tumors) criteria for the follow-up period. This wis a globally recognized standard for evaluating a patient's tumor response to treatment, used to assess whether tumors shrank, stayed the same, or grew. It was the standard used by our medical team for years, which made it impractical to apply another criterion retroactively. The low mortality rate observed in our study was because all patients were in Stage II. Stage II cancer was considered an early stage and was often curable with treatments like surgery, radiation, or chemotherapy, which was a reason for the lower mortality rate. Our study only considered deaths from cancer, and we did not have access to information on deaths from other causes.
