# Supplementary material for: Hidden risks associated with occupational pesticide exposure in women with breast cancer: High frequency of the Luminal B molecular subtype and occurrence of poor prognostic features
Source: PLoS One. 2026 Feb 5;21(2):e0339471. doi: 10.1371/journal.pone.0339471 (PMC12875504; doi:10.1371/journal.pone.0339471)
Supplement: Attachment — Submitted filename: 03-Response_to_Reviewers_5_2025-10-28.docx [file pone.0339471.s009.docx]

PONE-D-24-40245R2

Hidden risks associated with occupational pesticide exposure in women with breast cancer: high frequency of the Luminal B molecular subtype and occurrence of poor prognostic features.

Dear Dr. Sauli.

We would like to thank you again for your analysis, criticism and considerations. Below are the requested modifications.

Editor comments:

The authors should ASAP address raised methodological issues....or at least list them as limitations (in detail) if they can't address them.

Reviewer #9:

1. If the authors have adequately addressed your comments raised in a previous round of review and you feel that this manuscript is now acceptable for publication, you may indicate that here to bypass the “Comments to the Author” section, enter your conflict of interest statement in the “Confidential to Editor” section, and submit your "Accept" recommendation.

Correct response: The authors did not adequately implement the previous suggestions

In the first review, I highlighted several points that needed modification, considering major revisions.

In the second version, the changes were not made, and the points that needed improvement were included, and the article was rejected due to inadequate data.

Now, in the third version, the suggested points were not changed, so I must maintain the rejection finding.

The problem is methodological. It lists four breast tumor subtypes, and we have five. It describes recurrence without describing adequate follow-up. Unaddressed methodological issues prevent the article from being accepted.

R.: We sincerely apologize for this. We understand that there are different evaluation criteria, both for patient follow-up and for the classification of molecular subtypes, the two main points indicated by the last reviewer of the paper. We believe that these criteria are gradually modified over the years by the scientific community and that, therefore, different interpretations are possible, especially in a complex pathology such as breast cancer. Thus, we agree that it is necessary to clearly and objectively include the criteria that were used in the study, which we have sought to do in this last revision. We also believe that the work has already been thoroughly evaluated by eight different reviewers and that the contribution of the last reviewer, even if they do not agree with our criteria, can bring more clarity to the current version of the article.

Details about the follow-up period and recurrence assessment have been added in the revised version of the manuscript.

Methods section , Pag 9 and 10

“Imaging reports from mammography, diagnostic ultrasound, breast MRI, CT, and PET-CT during follow-up were analyzed by clinicians in accordance with RECIST recommendations to establish baseline and post-treatment status. For each patient, the same imaging modality was applied at baseline and follow-up to ensure consistency.”

“Treatment response was categorized as follows: complete response, defined as the disappearance of all target lesions; partial response, defined as at least a 30% reduction in target lesion size without evidence of new lesions; progressive disease, defined as a minimum 20% increase in lesion size relative to baseline and/or the appearance of new lesions in the breast or distant organs; and stable disease, defined as insufficient shrinkage to meet partial response criteria and insufficient increase to meet progression criteria. Patients were monitored over a five-year follow-up period. Based on these criteria, patients were classified as responsive (complete response) or chemoresistant (partial response, progressive disease, or stable disease).”

“We used only the RECIST (Response Evaluation Criteria In Solid Tumors) criteria for the follow-up period. This wis a globally recognized standard for evaluating a patient's tumor response to treatment, used to assess whether tumors shrank, stayed the same, or grew. It was the standard used by our medical team for years, which made it impractical to apply another criterion retroactively. The low mortality rate observed in our study was because all patients were in Stage II. Stage II cancer was considered an early stage and was often curable with treatments like surgery, radiation, or chemotherapy, which was a reason for the lower mortality rate. Our study only considered deaths from cancer, and we did not have access to information on deaths from other causes.”

We have now included a detailed explanation of the separation of molecular subtypes as a study limitation, acknowledging the absence of Luminal-HER2 cases as a factor that may restrict the generalizability of our findings, as follows:

“In previous classifications, the Luminal B subtype of breast cancer was often defined as estrogen receptor (ER)-positive and HER2-amplified, meaning that tumors expressing concomitant hormone receptors and HER2 overexpression were categorized as Luminal B (HER2-positive). Currently, the Luminal B subtype is identified independently of HER2 status, being characterized instead by positive hormone receptor expression (ER and/or PR) combined with a high proliferative index (Ki-67 >14%), regardless of HER2 amplification. The initial design of our study did not aim to include patients with the Luminal-HER2 subtype due to the small number of cases in this subgroup; therefore, this subgroup was excluded from the final analysis to avoid biased interpretations based on an insufficient sample size. Since their data were not collected, they could not be included in the present study. The results were presented considering only the four main breast cancer subtypes, considering the St. Gallen International Expert Consensus (Figure XXX), which recognizes that molecular classification based on immunohistochemistry groups breast tumors into these four main categories. We believe this decision does not compromise the methodological rigor or the scientific merit of our study (Coates 2015, Burstein 2023).”

Table XXX - Definition of subtypes of breast cancer included in the study accordingly to the St. Gallen classification (Goldhirsch et al., 2013)

| Subtypes of breast cancer | ER and PR | HER2 | Ki67 |
| --- | --- | --- | --- |
| Luminal A | ER + and/or PR + | negative | Ki-67<14% |
| Luminal B | ER+ and/or PR+ | negative | Ki-67≥14% |
| HER2 enriched | ER-, PR- | positive | Any Ki-67 |
| Triple negative | ER-, PR- | negative | Any Ki-67 |

Legend: negative (-), positive (+). Luminal-HER2 patients (ER+, PR+, and HER2+) were not included in the study.

A. Goldhirsch, E.P. Winer, A.S. Coates, R.D. Gelber, M. Piccart-Gebhart, B. Thürlimann, H.-J. Senn, Kathy S. Albain, Fabrice André, Jonas Bergh, Hervé Bonnefoi, Denisse Bretel-Morales, Harold Burstein, Fatima Cardoso, Monica Castiglione-Gertsch, Alan S. Coates, Marco Colleoni, Alberto Costa, Giuseppe Curigliano, Nancy E. Davidson, Angelo Di Leo, Bent Ejlertsen, John F. Forbes, Richard D. Gelber, Michael Gnant, Aron Goldhirsch, Pamela Goodwin, Paul E. Goss, Jay R. Harris, Daniel F. Hayes, Clifford A. Hudis, James N. Ingle, Jacek Jassem, Zefei Jiang, Per Karlsson, Sibylle Loibl, Monica Morrow, Moise Namer, C. Kent Osborne, Ann H. Partridge, Frédérique Penault-Llorca, Charles M. Perou, Martine J. Piccart-Gebhart, Kathleen I. Pritchard, Emiel J.T. Rutgers, Felix Sedlmayer, Vladimir Semiglazov, Zhi-Ming Shao, Ian Smith, Beat Thürlimann, Masakazu Toi, Andrew Tutt, Michael Untch, Giuseppe Viale, Toru Watanabe, Nicholas Wilcken, Eric P. Winer, William C. Wood. Personalizing the treatment of women with early breast cancer: highlights of the St Gallen International Expert Consensus on the Primary Therapy of Early Breast Cancer 2013, Annals of Oncology, Volume 24, Issue 9, 2013, Pages 2206-2223, ISSN 0923-7534, https://doi.org/10.1093/annonc/mdt303.

Coates, A. S., Winer, E. P., Goldhirsch, A., et al. (2015). Tailoring therapies—improving the management of early breast cancer: St. Gallen International Expert Consensus on the Primary Therapy of Early Breast Cancer 2015. Annals of Oncology, 26(8), 1533–1546.

Burstein, H. J., Curigliano, G., Thürlimann, B., et al. (2023). Customizing therapies for women with early breast cancer: St. Gallen International Consensus Conference 2023. Annals of Oncology, 34(7), 573–592.
