# Supplementary material for: Hidden risks associated with occupational pesticide exposure in women with breast cancer: High frequency of the Luminal B molecular subtype and occurrence of poor prognostic features
Source: PLoS One. 2026 Feb 5;21(2):e0339471. doi: 10.1371/journal.pone.0339471 (PMC12875504; doi:10.1371/journal.pone.0339471)
Supplement: Attachment — Submitted filename: 03-Response_to_Reviewers_6_2025-12-04.docx [file pone.0339471.s010.docx]

PONE-D-24-40245R2

Hidden risks associated with occupational pesticide exposure in women with breast cancer: high frequency of the Luminal B molecular subtype and occurrence of poor prognostic features.

Dear Dr. Sauli.

We would like to thank you again for your analysis, criticism and considerations. Below are the requested modifications.

Editor comments:

THE AUTHORS SHOULD ASAP ADDRESS RAISED METHODOLOGICAL ISSUES (by Reviewer 9 AS LIMITATIONS, IF THEY CANT BE SOLVED AS QUERIED. THIS SHOULD BE DONE ASAP BEFORE THIS SUBMISSION CAN BE ACCEPTED.

R.: Regarding your recent decision about our manuscript, we would like to state clearly that all revisions previously requested by Reviewer #9 were fully and rigorously implemented in the current version of the manuscript. These modifications are documented both in our detailed response letter and in the tracked-changes version of the manuscript.

Reviewer #9 stated that the authors did not adequately implement the previous suggestions and that methodological concerns remain unresolved.

We respectfully disagree with this assertion. Every point raised by Reviewer #9 in the earlier round was directly and comprehensively addressed. The current version includes all required corrections which the authors understand as an improvement to the work, clarifications, and methodological details. The aspects that were not modified, given that differences in interpretation may occur due to the evolution of clinical criteria and the different perspectives of experts in breast cancer research, were indicated as limitations of the study. However, these differences do not indicate a lack of revision..

Detailed descriptions of the follow-up period, recurrence definitions, imaging protocols, and RECIST-based response criteria were added to the Methods section (pages 9–10), exactly as previously requested. The rationale for the low mortality rate and the exclusive inclusion of Stage II patients was explicitly explained. The classification of molecular subtypes was fully clarified, including the methodological decision to exclude Luminal-HER2 cases due to insufficient sample size. Also, a limitation was added acknowledging this exclusion, along with a detailed table defining all subtypes according to the St. Gallen criteria. We would also like to highlight that although we acknowledge the existence of five molecular subtypes of breast cancer, our study includes data for only four of them. Unfortunately, we did not have enough Luminal–HER2 subtype samples available for inclusion, and we have fully addressed this constraint as a study limitation in the revised manuscript.

Therefore, all revisions requested by Reviewer #9 are now present, explicit, and traceable throughout the manuscript. In light of this, we would like to kindly emphasize that if the reviewer our the editor believes any additional, specific information is still missing, we respectfully request that such a point be indicated clearly and directly so that we can address it precisely. Since the last three comments we received from the editor contained the same requests, we were left with some doubt, given that the modifications had already been discussed.

General statements suggesting that revisions were “not made” do not reflect the content of the updated manuscript, which fulfills every requirement previously listed. We also highlight that the manuscript has already undergone evaluation by eight independent reviewers, who fully approved the work, and that the revisions made, including those prompted by Reviewer #9, substantially strengthened its methodological transparency.

In light of these comprehensive adjustments, we firmly maintain that the concerns raised by Reviewer #9 have been fully addressed and that the manuscript is methodologically sound and suitable for publication.

Confident in your understanding, we thank you and remain at your disposal.
